# Robust COVID-19 vaccination response after allogeneic stem cell transplantation using post transplantation cyclophosphamide conditioning

**DOI:** 10.1038/s41408-021-00605-1

**Published:** 2022-01-12

**Authors:** L. M. Morsink, J. van Doesum, G. Choi, C. L. E. Hazenberg, A. Biswana, F. Meppelink, L. B. Bungener, A. J. A. Lambeck, G. Huls

**Affiliations:** 1 Department of Hematology, University Medical Center Groningen, Groningen, the Netherlands; 2 University of Groningen, Groningen, the Netherlands; 3 Department of Laboratory Medicine, University Medical Center Groningen, Groningen, the Netherlands

**Keywords:** Bone marrow transplantation, Acute myeloid leukaemia

**Dear Editor**,

Since the rise of the COVID-19 pandemic, clinicians aim to understand the implications of this new infectious disease, enabling them to make evidence based clinical decisions for their specific patient populations. Despite increasing knowledge on the antibody response of SARS-CoV-2 vaccinations in hematological patients and more specifically in patients treated by means of an allogeneic stem cell transplantation, limited data are available on the impact of treatment with cyclophosphamide post transplantation (PTCy). Cyclophosphamide exerts its effect through specific deletion of proliferating alloreactive T-cells on day +3 and +4 after stem cell infusion, leaving the T-cell compartment responsible for immune reconstitution and resistance to infection, largely unaffected [1]. We hypothesize that this might preserve the ability to mount a robust antibody response against SARS-CoV-2, even in case of vaccination early after allogeneic stem cell transplantation.

In this single-center, retrospective analysis, we describe a cohort of 70 consecutive patients transplanted for acute myeloid leukemia (AML) between February 2017 and July 2021, who all received PTCy containing conditioning. All patients were vaccinated with at least one dose of any type of SARS-CoV-2 vaccine, with the majority of patients receiving their first vaccination in April or May 2021. Antibody responses were determined as standard of care in September 2021 using the Abbott SARS-CoV-2 IgG EUA assay. A cut-off level of 300 arbitrary units (AU)/ml was chosen based on a recent analysis showing the association between SARS-CoV-2-binding antibody concentration (BAU/ml) and neutralizing antibody titer that categorized responders into adequate responders (> 300 BAU/mL) and suboptimal responders (> 10 BAU/mL but ≤300 BAU/mL) [2]. The antibody status of patients prior to vaccination was unknown. Previous exposure to endemic (seasonal) coronaviruses was also not documented [3].

Applied conditioning prior to transplantation was guided by age; generally, patients younger than 60 years received either 12 Gy total body irradiation (TBI) or busulfan-based conditioning (dose 0.8 mg/kg/AIBW QID) on 4 consecutive days (Bu4); patients aged 60–70 years received either 8 Gy TBI or Bu3; patients older than 70 years received Bu2 (see Table 1 for details). Standard graft versus host prophylaxis consisted of tacrolimus and MMF for haplo- or mismatched MUD transplantations (stop MMF at day +28 to +35, stop tacrolimus at day +56 to +70) and tacrolimus in matched donor transplantations (stop tacrolimus at day +28 for patients transplanted with active disease or MRD positivity, otherwise stopped at day +70).

**Table 1:** Patient and treatment characteristics.

	Transplanted PTCy AML patients (*n* = 70)
Median time between HCT and first vaccination (months) (range)	27.8 (0.6–49.5)
Median age at HCT (range), years	62 (24–76)
Male sex, *n* (%)	41 (59)
Disease status at HCT, *n* (%)	
Active disease	7 (10)
CR1(MRDpos)	37 (53)
CR1(MRDneg)	21 (30)
CR1(no LAP)	3 (4)
CR2(MRDpos)	2 (3)
Type of induction therapy, *n* (%)	
Intensive treatment	35 (50)
HMA	10 (14)
Combination of intensive and HMA	25 (36)
Donor type, *n* (%)	
SIB	10 (14)
MUD 10/10	47 (67)
MMUD 9/10 or 8/10	4 (6)
HAPLO	9 (13)
Conditioning regimen, *n* (%)	
6.6–12 Gy (Flu + ) TBI + PTCy (MAC)	34 (49)
3/4 Bu + Flu + PTCy (MAC)	13 (19)
2/3 Bu + Flu + Thio + PTCy (MAC)	2 (3)
1–2 Bu + Flu + PTCy (RIC)	16 (23)
1 Bu + Flu + Thio + PTCy (RIC)	3 (4)
2 Gy TBI + Cy + Flu + PTCy (RIC)	2 (3)
Stem cell source, *n* (%)	
PBSC	61 (87)
BM	9 (13)
CD34+ number transplanted × 10^6^/kg, mean, (range)	
PBSC	8.0 (1.9–14.9)
BM	4.1 (1.6–7.4)
CD3+ number transplanted × 10^6^/kg, mean, (range)	
PBSC	306 (134–826)
BM	30.8 (20.8–51.4)
GVHD prophylaxis, *n* (%)	
Tacrolimus/MMF	13 (19)
Tacrolimus	57 (81)
Using IS at time of first vaccination, *n* (%)	14 (20)
Tacrolimus	7 (10)
Tacrolimus and/or low dose prednisolone	4 (6)
Ruxolitinib	2 (3)
Ibrutinib and tacrolimus	1 (1)
Median time between discontinuation of IS and 1st vaccination, (months (range)	12.4 (0.1–44.1)
Type of vaccination, *n* (%)	
mRNA-1273	54 (77)
BNT162b2	8 (11)
Combination mRNA-1273/BNT162b2	6 (9)
Other	2 (3)
Prior COVID infection, *n* (%)	
Mild	6 (9)
Severe (requiring hospital admission)	1 (1)

*BM* bone marrow, *Bu* busulfan, *CR* complete remission, *Cy* cyclophosphamide, *Flu* fludarabine, *GVHD* graft versus host disease, *HCT* hematopoietic cell transplantation, *HMA* hypomethylating agent, *IS* immune suppression, *LAP* leukemia associated phenotype, *MAC* myeloablative conditioning, *MRD* measurable residual disease, *MMUD* mismatched unrelated donor, *MUD* matched unrelated donor, *PBSC* peripheral blood stem cell, *PTCy* post transplantation cyclophosphamide, *RIC* reduced intensity conditioning, *SIB* sibling, *TBI* total body irradiation, *Thio* thiotepa.

Characteristics of the analyzed patient cohort are depicted in Table 1. Within this cohort of 70 patients, 54 patients (77%) were vaccinated with mRNA-1273 (Moderna), 8 patients (11%) with BNT162b2 (Pfizer BioNTech), 6 patients (9%) received both and 2 (3%) were vaccinated with ChAdOx1 nCoV-19 (Astra Zeneca). Three patients, who had been diagnosed with a symptomatic COVID infection prior to initiation of the vaccination program, received only one vaccination (all BNT162b2). Median time between transplantation and administration of the first vaccination was 27.8 months (range 0.6–49.5 months).

**Fig. 1:**
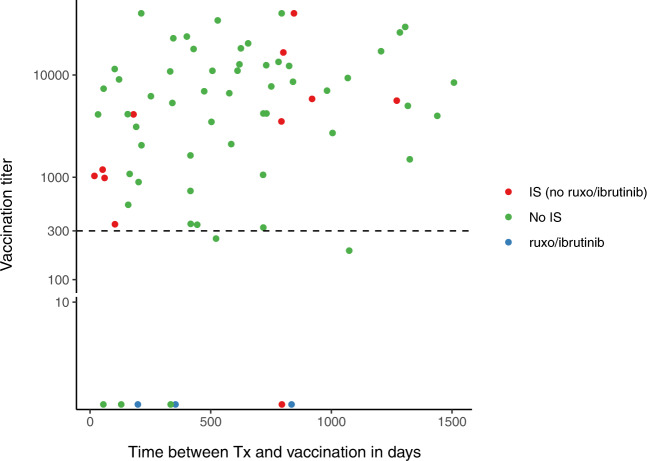
Relationship between antibody titer after COVID vaccination and time after allogeneic stem cell transplantation with post transplantation cyclophosphamide containing conditioning. Vaccination titer in AU/ml.

The large majority of our cohort, 61 patients (87%), developed an adequate antibody response (titer > 300 AU/ml) and 53 patients (76%) had robust responses with a titer >1000 AU/ml (see Fig. 1).

Nine patients (13%) did not have a sufficient response to vaccination, of whom 2 patients were low responders (titer 0 ≤ 300 AU/ml) and 7 patients were non-responders (titer non-detectable). Of the 7 non-responders, 4 were treated with immune suppression for chronic graft versus host disease (cGVHD) (1 patient with tacrolimus and ibrutinib, 1 with tacrolimus and low dose prednisone and 2 with ruxolitinib monotherapy) at the time of vaccination. Two of the non-responders had stopped immunosuppressive medication 20 and 60 days before vaccination.

Of the patients with an adequate antibody response, 10 were being treated with tacrolimus and/or low dose of prednisolone at time of vaccination. All patients using ruxolitinib or ibrutinib were non-responders (*n* = 3). Within our cohort, 10 patients were vaccinated soon after transplantation (between 18 and 120 days, with 4 patients still using tacrolimus). Only 1 of these 10 patients, who received ChAdOx1 nCoV-19 vaccination, appeared to be a non-responder.

This homogeneous cohort of AML patients who received an allogeneic stem cell transplantation with PTCy conditioning, shows that the majority of patients has a robust and prolonged (>5 months after 1st vaccination) antibody response after vaccination with an anti-SARS-CoV-2 mRNA vaccine. Although the patient numbers are small, our data show that vaccination shortly after allogeneic stem cell transplantation with concurrent GVHD prophylaxis with tacrolimus, results in sufficient antibody responses (see Fig. 1). This is in line with the general vaccination recommendations by the ECIL 7 guidelines, which recommend to start the revaccination program 3 months after transplantation, since sufficient immune reconstitution is to be expected to induce an adequate response to vaccination [4].

Our results are comparable with a Lithuanian cohort of 122 patients undergoing an allogeneic stem cell transplantation, with 85% having finished their last treatment >12 months before first vaccination. Mean antibody titer was 6304 AU/mL (1120–16,913) 7–21 days after second immunization. Type of conditioning or ongoing use of immune suppression was not reported [5].

In another study cohort, a sufficient humoral response to Pfizer vaccination was found in 68% of allogeneic stem cell transplantation patients, in comparison to a response rate of 100% in health care workers used as control [6]. Details concerning transplant regimens, the use of PTCy specifically and treatment with immune suppression were again not available.

In line with our observations, the Lithuanian study cohort also showed that patients treated with ruxolitinib and ibrutinib both had very poor responses (Bruton tyrosine kinase inhibitors; *n* = 44; 0 AU/mL [0–7] and ruxolitinib; *n* = 16; 10 AU/mL [0–45]) [5].

It remains unclear whether these adequate vaccination responses after transplantation are a result of a specific pattern of immune reconstitution related to the use of PTCy, allowing a relative early cessation of immune suppression or that these responses can also be achieved in patients transplanted with different conditioning strategies (ATG or alemtuzumab-based T-cell depletion or no T-cell depletion with prolonged use of multiple immunosuppressive agents). The data obtained in our cohort provide valuable information to advise patients how PTCy impacts on the efficacy of vaccinations against SARS-CoV-2.

This retrospective analysis of a consecutive patient cohort shows that AML patients, after allogeneic stem cell transplantation using PTCy conditioning, show a robust immune response to a SARS-CoV-2 mRNA-vaccination, even when administered relatively early after transplantation while still using tacrolimus.
